# Biomedical Applications of Supramolecular Materials in the Controllable Delivery of Steroids

**DOI:** 10.3389/fmolb.2021.700712

**Published:** 2021-07-23

**Authors:** Yun Hao, Feiyi Zhang, Shanshan Mo, Jinming Zhao, Xiangdong Wang, Yan Zhao, Luo Zhang

**Affiliations:** ^1^ Department of Otolaryngology Head and Neck Surgery, Department of Allergy, Beijing TongRen Hospital, Capital Medical University, Beijing, China; ^2^ Beijing Key Laboratory of Nasal Diseases, Beijing Institute of Otolaryngology, Beijing, China; ^3^ Institute for Advanced Materials, Jiangsu University, Zhenjiang, China; ^4^ State Key Laboratory of Medical Molecular Biology, Institute of Basic Medical Sciences Chinese Academy of Medical Sciences, School of Basic Medicine Peking Union Medical College, Beijing, China; ^5^ Chinese Academy of Medical Sciences and Peking Union Medical College, Beijing, China

**Keywords:** steroids, supramolecular materials, interaction, biomedical application, chronic rhinosinusitis

## Abstract

Glucocorticoids are a class of steroid hormones secreted from the adrenal glands. The strong anti-inflammatory effects make it be one of the most popular and versatile drugs available to treat chronic inflammatory diseases. Additionally, supramolecular materials have been widely exploited in drug delivery, due to their biocompatibility, tunability, and predictability. Thus, steroid-based supramolecular materials and the release of steroids have been applied in the treatment of inflammatory diseases. This mini-review summarized recent advances in supramolecular materials loaded with glucocorticoid drugs in terms of hydrophobic interactions, electrostatic interactions, hydrogen bonding, and π-π stackings. We also discussed and prospected the application of the glucocorticoid drugs-based supramolecular system on chronic rhinosinusitis, multifactorial inflammatory disease of the nasal and paranasal sinuses mucosal membranes. Overall, supramolecular materials can provide an alternative to traditional materials as a novel delivery platform in clinical practice.

## Introduction

Glucocorticoids belong to the family of cholesterol-derived hormones produced by the adrenal glands. They have been discovered and applied into clinical medicine in the 1940s (Vandewalle et al., 2018) and can be administered orally, intravenously, or topically (Luhder and Reichardt, 2017). Considering their strong and efficient anti-inflammatory effects, glucocorticoids has been generally introduced to the treatment of rheumatoid arthritis (Aletaha and Smolen, 2018), asthma (Barnes, 2011), atopic dermatitis (Mayba and Gooderham, 2017), allergic rhinitis (Wheatley and Togias, 2015) and chronic rhinosinusitis (Hopkins, 2019). However, the side effects of glucocorticoids cannot be ignored. They can arise hypothalamic-pituitary-adrenal suppression-related complications such as hyperglycemia, aseptic necrosis of the femoral head, negative calcium balance, mood disorders, and Cushing’s syndrome (Chotiyarnwong and McCloskey, 2020). It is urgent to develop a novel therapeutic regimen for steroids delivery and release.

Traditional chemistry used in the synthesis and application of glucocorticoids focuses on the covalent bond; however, supramolecular chemistry mainly examines the weaker and reversible non-covalent interactions such as hydrophobic, electrostatic, hydrogen bonding, and π-π stackings (Mendes et al., 2013). In general, molecules self-assembled by non-covalent interactions in specific solvent and formed supramolecular materials (Zhang et al., 2020a). Supramolecular materials have biocompatibility, tunability, and predictability (Zhou et al., 2017; Yu et al., 2020). Thanks to the advantages of supramolecular materials, significant advances have been made in drug delivery. Glucocorticoid drugs have many characteristics for loading onto supramolecular materials. Firstly, their typical hydrophobic structure promotes the combination with amphiphilic supramolecular materials. Secondly, the incorporation of anionic groups assists in combining with materials through electrostatic interactions. Lastly, their ordered structure and abundant π electrons indicate the existence of hydrogen bonds and π-π stackings.

In this review, we outlined the recent progress on basic theories and biomedical applications in supramolecular materials with glucocorticoid drugs via four kinds of interactions including hydrophobic interaction, electrostatic interaction, hydrogen bond, and π-π stackings. We hope our review would provide new ideas for the application of steroids-based supramolecular materials in the treatment of more diseases.

## Hydrophobic Interactions

Hydrophobic interaction is a tendency of nonpolar substances to aggregate in an aqueous solution, which increases hydrogen bonds between water molecules and decreases the area of contact between water and nonpolar molecules. Distinct from electrostatic interactions and hydrogen bonds, hydrophobic interactions can be elucidated as a thermodynamic effect with changes in free energy, entropy, enthalpy, and heat capacity rather than one of the fundamental types of molecular interactions (Shen et al., 2017; Yadav et al., 2020). When hydrophobic groups of molecules contact with water molecules, the entropic effect leads to a rearrangement of water molecules. Researchers have increasingly focused more attention on biomaterials to make the best use of hydrophobic interactions. It is commonly observed that without applying amphiphilic materials or combining with hydrophilic molecules, hydrophobic drugs alone fail to self-assemble to nanostructures (Wang et al., 2012). Therefore, some bonds such as disulfide, thioether, ester bonds, or amphiphilic materials were added to the hydrophobic drugs to self-assemble into nanostructures in aqueous solutions (Wang et al., 2014).

**FIGURE 1:**
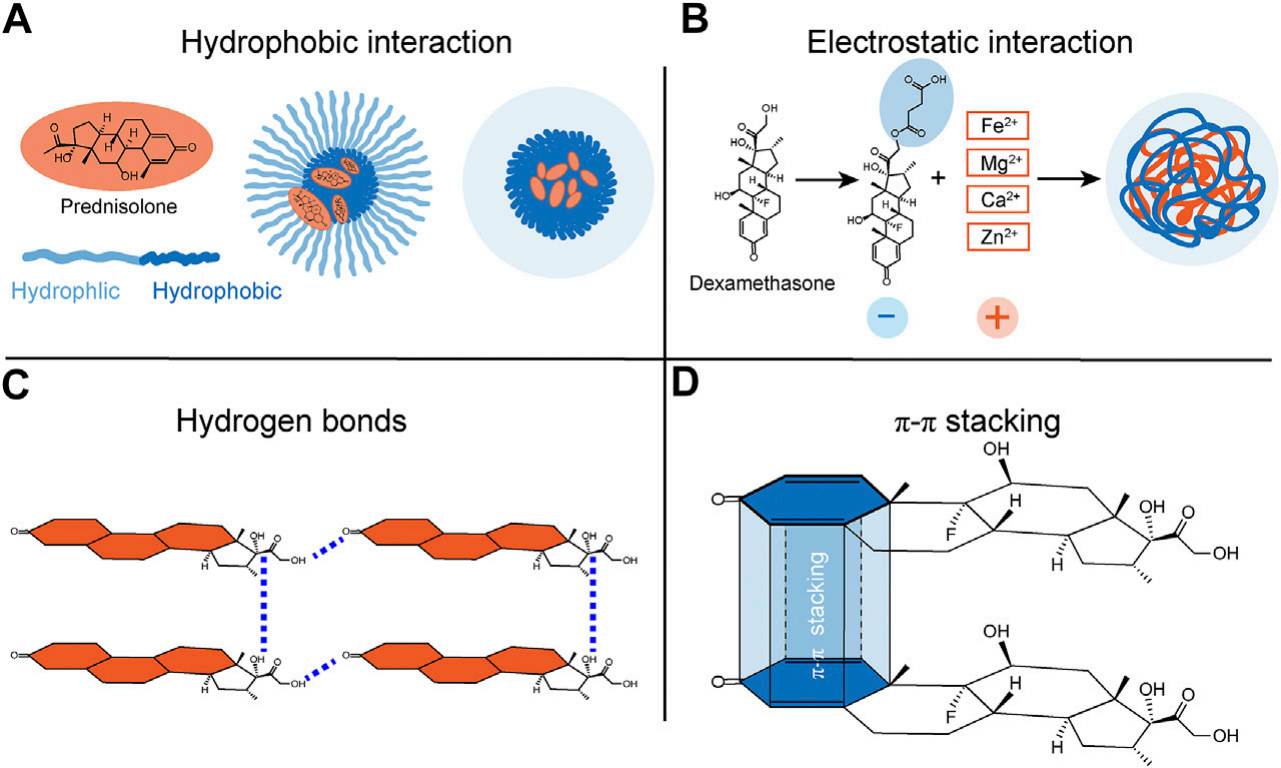
Schematic representations of possible interactions between supramolecular materials and steroids. The supramolecular materials bind to steroids possibly via **(A)** hydrophobic interactions, **(B)** electrostatic interactions, **(C)** hydrogen bonds, and **(D)** π-π stacking interactions.

It has been demonstrated that the glucocorticoids could attenuate inflammations in acute lung injury (Tu et al., 2017), arthritis (Aletaha and Smolen, 2018), and atherosclerosis (van der Sluis and Hoekstra, 2020). Compared to normal cells, inflammatory cells tend to overexpress reactive oxygen species (ROS), accompanied by the inflammatory process. Hence, ROS-responsive nanoparticles have been introduced as targets for precise drug release treatment in inflammatory diseases. Ma et al. (Ma et al., 2020) reported that a ROS-responsive linkage was used to bridge the prednisolone and two-photon fluorophore (TP), constituting a diagnosis-therapy compound named TPP. Adding TP to prednisolone promoted the hydrophobic interaction of steroids obviously and provided a dimensional location for inflammation. The compound TPP was then packaged with an amphipathic diblock copolymer poly (2-methacryloyltoxyethyl phosphorylcholine)-poly (2-methylthio ethanol methacrylate) (PMPC-PMEMA) via hydrophobic interaction. The compound TPP and amphipathic copolymer PMPC-PMEMA were dissolved in the DMSO and methanol solution with phosphate-buffered saline (PBS) was added dropwise, stirred, and filtered. Afterward, the self-assembled form was obtained. With PMPC serving as a hydrophilic segment and PMEMA as a hydrophobic block, PMPC-PMEMA exhibited great superiority of protein adsorption resistance, biocompatibility, and responsiveness to ROS. The results showed that the particle size increased and prednisolone was released with the increased concentration of H_2_O_2_, an oxidizer that induces reactive ROS formation (Ogawa et al., 2004). Prednisolone was slowly released without the stimulation of H_2_O_2_, while it showed rapid release under the H_2_O_2_ effect. After 48 h, there was approximately 89% of prednisolone delivery. After being treated with H_2_O_2_ for 4 h, noncompact micelles started to aggregate due to the hydrophobic interaction of TP, suggesting the outstanding reversibility of these supramolecular materials (Figure 1A).

In recent years, some *in vivo* disease models have been established to evaluate the effectiveness of the supramolecular materials loaded with glucocorticoids. They found that the supramolecular materials loaded with glucocorticoids treatment could significantly inhibit the pulmonary edema in the acute lung injury mice model than saline group. In addition, the supramolecular materials loaded with glucocorticoids treatment also exhibited a more detumescence effect than the untreated group in the collagen-induced arthritic joints model. Furthermore, it could strongly reduce oxidized low-density lipoprotein uptake and inhibit foam cell formation for atherosclerosis *in vitro* and *in vivo*. The outstanding theragnostic of inflammation for the glucocorticoid drug delivery system has been proved in the treatment of acute lung injury, arthritis, and atherosclerosis (Ma et al., 2020).

Moreover, Chung et al. Chung et al. (2020) reported the synthesis of another supramolecular material combined with steroids. Poly (ethylene glycol) (PEG) played a hydrophilic role, while rosmarinic acid (RA), an anti-inflammatory and anticancer component, played a strong hydrophobic role. They were prepared and self-assembled, relying on hydrophobic interactions when dexamethasone was incorporated into the solution. Steroid-based hydrogel treatment could recover colon length dose-dependently in the dextran sulfate sodium salt (DSS) induced colitis mice model (Chung et al., 2020).

**FIGURE 2:**
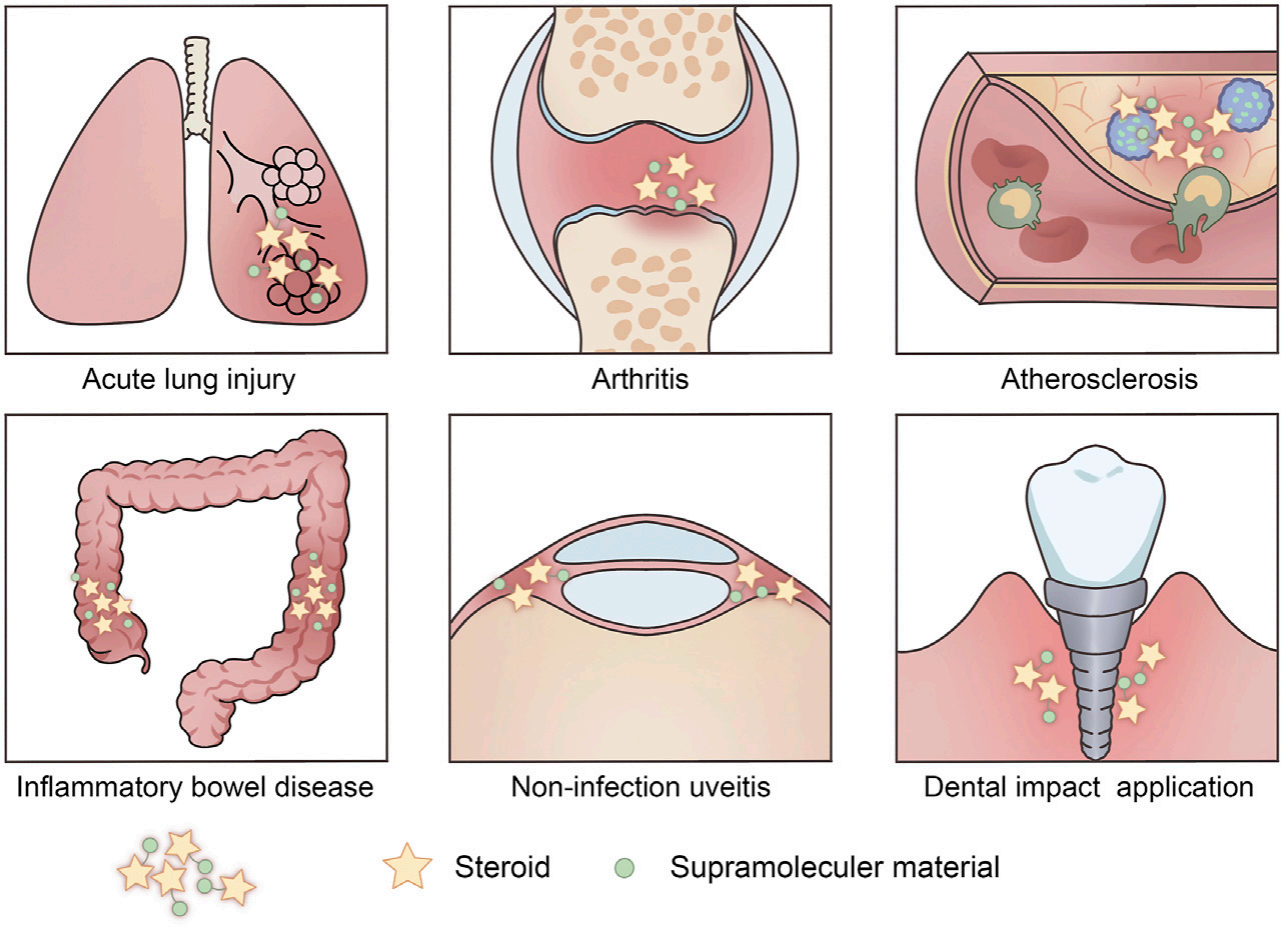
The biomedical applications of the glucocorticoid drug delivery systems. Glucocorticoid drugs based supramolecular systems have been widely used in acute lung injury, arthritis, atherosclerosis, inflammatory bowel disease, non-infection uveitis, and dental impact application.

Generally, hydrophobic interaction is a common non-covalent interaction widely applied to various fields. As for steroids, a kind of prominent hydrophobic drug, hydrophobic interaction with supramolecular materials might be a trend in the future of drug delivery (Figure 2; Table 1).

**TABLE1:** Features and medical applications of different supramolecular interactions used for the delivery of steroids.

Interactions		Medical applications	References (PMID)
Hydrophobic interaction	Ma et al. (2020)	Acute and chronic inflammation	PMID: 32379416
	Chung et al. (2020)	Acute inflammatory bowel disease	PMID: 32449857
Electrostatic interactions	Zhou et al. (2018)	Non-infectious uveitis	PMID: 29803956
	Wu et al. (2017)	Non-infectious uveitis	PMID: 28501709
Hydrogen bond	Fraile et al. (2016)	Posterior segment ocular diseases	PMID: 27594212
	Wu et al. (2017)	Non-infectious uveitis	PMID: 28501709
π-π stacking interaction	Jung et al. (2015)	Dental implant applications	PMID: 25909563
	Wu et al. (2017)	Non-infectious uveitis	PMID: 28501709

## Electrostatic Interactions

Electrostatic interactions are long-range non-covalent interactions between two charged ions or molecules, occurring when ions or molecules with opposite charges attract each other (Schneider, 2009). Changes in the pH and ionic strength of the solution can moderate the forces of electrostatic interactions (Shen et al., 2017). Electrostatic interactions have been used widely in some positively or negatively charged parts of the body. For instance, drugs encapsulated in cationic carriers could rapidly penetrate negatively charged cartilage (Bajpayee and Grodzinsky, 2017). Another drug was provided with a positive charge to bond with the negatively charged bladder mucosa to achieve sustained drug release (Guo et al., 2017). Contrary to the cartilage and bladder, the drug delivery problem that should be overcome in mucosal tissues is rapid removal by the mucus. Drugs can be trapped in mucosa via electrostatic or hydrophobic interactions (Dunnhaupt et al., 2015). A high density of positive and negative surface charges facilitates drug delivery through the mucosal layer by minimizing the electrostatic interactions with mucus.

As a typical case of the mucosa, in the field of non-infectious uveitis, Zhou et al. Zhou et al. (2018) designed and synthesized steroidal drug-based supramolecular hydrogels by electrostatic interactions and metal coordination. Firstly, a carboxylic acid group was added to dexamethasone to enhance electronegativity. Succinate dexamethasone (Dex-SA) was suspended in PBS, followed by incorporating Na_2_CO_3_ solution to provide a transparent solution. Next, due to the incorporation of various cations (Mg^2+^, Ca^2+^, Zn^2+^, Fe^2+^, Cu^2+^, and NH_4_^+^), Dex-SA self-assembled to form steroidal supramolecular hydrogels. Besides, the results showed distinct gelation ability among cations in the order of Mg^2+^>Ca^2+^>Zn^2+^≈Fe^2+^˃NH_4_^+^. Divalent cations exhibited strong gelation ability due to two sites coordinating with a carboxylic acid. The ability of various divalent cations to induce gelation might be affected by the radii of cations. Cations with smaller radii lead to a stronger ability for gelation. Among the above cations, Ca^2+^ ions are most widely distributed in humans; therefore, attention has been focused on the synthesis and application of the Ca^2+^-Dex-SA supramolecular hydrogel. This Ca^2+^-Dex-SA supramolecular hydrogel exhibited good drug delivery ability modulated by changes in the Ca^2+^ concentration. Within 6 h, almost all Dex-SA and dexamethasone were completely released from the hydrogel. In addition, Ca^2+^-Dex-SA supramolecular hydrogel showed good reversibility upon large-amplitude oscillatory shear cycle. With an increase in Ca^2+^ concentration, the drug release rate slowed down, suggesting that the supramolecular material is Ca^2+^ responsive.

In addition, steroid-based supramolecular materials might be very effective for inflammation of non-infection uveitis. The non-infection uveitis rabbit model was induced by the carrageenan injection to increase epithelial thickness and the appearance of a horny superficial layer on the corneal surface. The triamcinolone acetonide incorporated into micellar solution treatment could improve the histological architecture of the cornea and nearly normal epithelial pattern for the corneal tissue (Safwat et al., 2020). With the rapid development of nanotechnology, the more and more extensive biological application of steroid-based supramolecular materials has been achieved (Figure 2; Table 1). And the widespread distribution of Ca^2+^ ions in the human body certainly provides us with a good prospect for further applications of supramolecular materials via electrostatic interactions (Figure 1B).

## Hydrogen Bonds

A hydrogen bond is an electrostatic interaction between the hydrogen with a partial positive charge atom or atomic group with a partial negative charge, where there is evidence of a bond formation. The electronegative atom directly connected with hydrogen bond by covalent bond is the hydrogen bond donor, while the hydrogen bond acceptor is the other electronegative atom which is not covalently attached to the hydrogen but participating in the formation of hydrogen bond. It is stronger than a van der Waals bond and weaker than fully covalent or electrostatic bonds. Of all the noncovalent bonds, a hydrogen bond is the one with the most pronounced directionality (Schneider, 2009). Some research teams synthesized layer-by-layer films employing hydrogen bonds between biologically compatible poly (acrylic acid) and a biodegradable block copolymer micelle to fully utilize the sensitive and directional characteristics of hydrogen bonds (Kim et al., 2008).

Concerning the use of hydrogen bonds on steroids, researchers dissolved dexamethasone and Ca^2+^ in distilled water to form dexamethasone supramolecular hydrogel. X-ray diffraction (XRD) analysis of dexamethasone supramolecular hydrogel suggested that the ordered structure exhibited a greater tendency to form hydrogen bonds and π-π stackings (Wu et al., 2017). The study speculation was reasonable, but no experimental verification was carried out to prove the formation of hydrogen bonds. The release of dexamethasone from hydrogel was categorized into a rapid release within the first 24 h and a sustained release in the subsequent 48 h. They also found that dexamethasone-based supramolecular hydrogel injection could significantly efficacy on the suppression of the uveal inflammatory response, while the saline group exhibited a severe inflammatory response in the anterior chamber accompanied by a large amount of purulent exudate (Wu et al., 2017) (Figure 2; Table 1).

Fraile et al. validated the formation of hydrogen bonds on dexamethasone through Fourier transform infrared (FT-IR) spectra and nuclear magnetic resonance (Fraile et al., 2016). They used laponite as a carrier for the controlled delivery of dexamethasone via hydrogen bonds. Dexamethasone was dissolved in ethanol or acetone, and laponite was added to this solution and stirred at room temperature; the solution self-assembled to form transparent colloidal dispersions. After removing the solvent, the whole amount of dexamethasone was deposited on the solid laponite. It is known that hydrogen bonds shift the X-H stretching frequency to a lower energy level (Feldblum and Arkin, 2014). The spectra results showed that, compared to the laponite alone group, the same C=O and C=C bands were slightly enlarged in the dexamethasone/laponite group, indicating that hydrogen bonds had formed in dexamethasone (Figure 1C). Furthermore, the carbonyl groups suffered a significant downfield shift in the ^1^H spectrum due to the decline in electronic density when accepting a hydrogen bond. The release of dexamethasone was based on the equilibrium between the physiosorbed and the dissolved forms of dexamethasone. The release of dexamethasone increased when its concentration was low. Self-assembled through hydrogen bonds, supramolecular materials loaded with steroid drugs will improve their responsiveness, biocompatibility, and tunability.

## π-π Stacking Interactions

A π-π stacking interaction refers to a type of noncovalent interaction involving unsaturated hydrocarbon predominantly containing π bonds (Wheeler, 2013). The π-π interaction can be categorized into T-shaped (edge-to-face stacked) and F-shaped (offset stacked and face-to-surface stacked), depending on the three-dimensional morphology of aromatic group interactions (Butterfield et al., 2002; Sinnokrot et al., 2002; Yu et al., 2020).

It was reported that supramolecular nanomaterials made of graphene had a honeycomb lattice framework where every carbon atom has its π electrons (Shim et al., 2017). The high density of π electrons makes it possible to use graphene-based nanomaterials for drug delivery via π-π interactions (Shim et al., 2016). Thus, there are quite a few aromatic drugs containing π electrons loaded on the graphene surface, suggesting that π-π stacking interactions play an outstanding role in designing chemical drug delivery systems (Lee et al., 2011).

In a study on multi-pass caliber-rolled Ti alloy of Ti1_3_Nb1_3_Zr (MPCR-TNZ) for dental implant applications, the high mechanical strength MPCR-TNZ was surface-modified with graphene oxide and loaded with the osteogenic drug dexamethasone. The weak strength of π-π interaction makes accurate measurements difficult in experiments (Zhuang et al., 2019). Considering the ordered structure in graphene and a large number of π electrons in graphene and dexamethasone, π-π stacking interactions could easily be speculated (Jung et al., 2015) (Figure 1D). *In vitro*, 10% of a total load of dexamethasone was released for a week, suggesting a long-term anti-inflammatory effect. The MPCR-TNZ loaded with dexamethasone showed facilitated differentiation of progenitor cells into osteoblasts compared to MPCR-TNZ material alone. Also, the dexamethasone-MPCR-TNZ group exhibited a significant amelioration in calcium-nodule formation and remarkable osteocalcin expression (Figure 2; Table 1).

Additionally, a study speculated the formation of π-π stacking and hydrogen bond interactions in dexamethasone supramolecular hydrogel (Wu et al., 2017). Although the application of π-π stacking interactions loaded with steroid drugs is relatively limited, π-π stacking interaction still has a good application prospect because of the ordered structure of steroids.

## Conclusions and Perspectives

This review discussed and summarized the hydrophobic interactions, electrostatic interactions, hydrogen bonds, and π-π stackings between supramolecular materials with steroid drugs and their biomedical applications. Understanding the basic theory of their hydrophobic groups, ordered structure and abundant π electrons clearly will help us to make more effective supramolecular materials-based steroid drugs.

At present, the steroid-based supramolecular systems have been widely used in acute lung injury, arthritis, atherosclerosis, inflammatory bowel disease, non-infection uveitis, and dental impact application. There are still some diseases that require better use of this steroid-based supramolecular system to develop new treatment strategies. For example, chronic rhinosinusitis (CRS) is a chronic heterogeneous disease that encompasses complex multifactorial inflammatory conditions of the nasal and paranasal sinuses mucosal membranes. According to current guidelines, intranasal glucocorticoids, systematic glucocorticoids, and functional endoscopic sinus surgery (FESS) are the principal therapeutic approaches for CRS (Fokkens et al., 2019). While only surgery may not control the persistent inflammation, it is essential to control postoperative inflammation and scarring, and these postoperative patients thus require ongoing oral or topical corticosteroids to reduce the inflammatory burden in the sinuses (Kohanski et al., 2018). In the recent decade, a new type of bioabsorbable steroid-eluting stent that engineered from polylactide-co-glycolide (PLGA) impregnated with steroid medication has emerged for CRS treatment (Murr et al., 2011; Han et al., 2014; Janisiewicz and Lee, 2015; Smith et al., 2016; Kern et al., 2018). The steroids were combined with PLGA materials using a physical coating method via dipping, spraying, brushing, or the layer-by-layer (LBL) assembly technology (Rykowska et al., 2020). However, there are some limitations in the application of PLGA. For example, the acidic by-products of PLGA can cause inflammation in the surrounding tissues. Additionally, the drug-eluting coronary stents reported that biodegradable stents (including PLGA stents) were associated with a higher stent thrombosis rate (Cassese et al., 2016). Also, an *in vitro* and *in vivo* study found prominent inflammation, eosinophil infiltration, and fibrotic tissue after stent implantation, indicating a prominent foreign-body reaction (Nikoubashman et al., 2018). Considering these limitations, specific research is necessary on promising materials to be utilized as a steroid-eluting stent. Owing to the properties in ordered superstructures, high responsiveness, biocompatibility, reversibility, tunability, and predictability, the combination of steroids and supramolecular materials may be expected to achieve sustained release as well as reduce the complications. Utilizing the responsiveness and reversibility, the self-assembly/disassembly processes of steroids can be controlled under chemical, physical, and biological stimuli.

Although supramolecular materials can be optimized to become a new generation of steroid drug delivery systems by skillfully adjusting these interactions. Nevertheless, several challenges limit the application of supramolecular materials containing steroids. Firstly, in most cases, the drug release time of supramolecular materials is less than that of macromolecular polymers. To achieve a long-lasting anti-inflammatory effect, prolonging drug release time will undoubtedly have a significant impact on optimizing supramolecular materials. The combination of supramolecular materials and macromolecular polymers might become a new drug delivery platform. Secondly, efforts are needed to develop bio-signaling-based release mechanisms. In tumors and some inflammatory diseases (Zhang et al., 2020b), the pH (Deirram et al., 2019), temperature (Zavgorodnya et al., 2017), redox potential (Raza et al., 2018), and even specific overexpressed proteins (Shigemitsu et al., 2020) could serve as stimulations to trigger the drug release of supramolecular materials. Besides, other steroid drugs and macromolecular drugs have been poorly developed in supramolecular materials (Geng et al., 2020). Some macromolecular drugs, such as the monoclonal antibodies, which play a specifically anti-inflammatory role in CRS, might fail to be delivered by supramolecular materials due to their large sizes. Further research is necessary for new strategies to recognize proteins through heteromultivalency or macrocycles with much larger cavity sizes for protein delivery.

Traditional drug delivery systems have widely been used in many fields of biomedicine. Moving forward, we anticipate that more supramolecular materials can replace traditional materials as novel delivery platforms in clinical practice.
